# Impacts of zinc oxide nano and bulk particles on redox-enzymes of the *Punica granatum* callus

**DOI:** 10.1038/s41598-020-76664-4

**Published:** 2020-11-12

**Authors:** Fatma A. Farghaly, Abeer A. Radi, Fatma A. Al-Kahtany, Afaf M. Hamada

**Affiliations:** 1 Botany and Microbiology Department, Faculty of Science, Assiut University, Assiut, 71516 Egypt; 2 Biology Department, Faculty of Science, Ibb University, Ibb, Yemen

**Keywords:** Plant sciences, Plant physiology

## Abstract

The structure and function of cellular membranes were sustained by redox-enzymes. We studied the interaction between the oxidative stress caused by excessive accumulation of ZnO-nanoparticles (ZnO-NPs) in plants and the role of redox-enzymes that can alleviate this stress. The crude callus extract from pomegranate, which was treated with 0, 10, and 150 µg mL^−1^ ZnO-NPs or bulk particles (ZnO-BPs), was applied to study the activity and kinetics of redox-enzymes. The elevated ZnO-NPs, enhanced the lipoxygenase and polyphenol oxidase activity, while the ZnO-BPs did not modify them. The activities of superoxide dismutase, catalase, and phenylalanine ammonia-lyase were induced under ZnO-NPs or BPs treatments, whilst the opposite trend of peroxidase was observed. Ascorbate peroxidase activity increased under ZnO-NPs treatments but decreased under ZnO-BPs. The kinetics activity of enzymes showed changes under different levels of NPs and BPs. Additionally, NPs or BPs treatments reduced the uptake of copper, iron, magnesium, but increased zinc accumulation in callus tissues. Meanwhile, these treatments enhanced the accumulation of manganese ions but did not affect the accumulation of potassium and phosphorous in ZnO-NPs or BPs-stressed calli. Collectively, these results gave a quantitative evaluation of the competition of zinc and other minerals on the carriers, and in addition, they provided a basis for how to control ZnO-NPs or BPs toxicity via redox-enzymes.

## Introduction

Nanotechnology has increased interest in many sciences, especially ZnO-NPs, which have received great attention due to their potential use in various sciences^1^. While there are broad fields of applications for ZnO-NPs, there is an interest in their release to ecosystems and their risks to resident organisms^2,3^. The release of ZnO-NPs is potentially toxic to organisms and reaches the food chain. Cytological tests of ZnO-NPs on pomegranate callus tissues showed that NPs reduced callus growth, dissociation of cytoplasmic content, and deformation of cell walls^4^.

In plants exposed to NPs, the uncontrolled output of reactive oxygen species (ROS) causes oxidative stress, which appears when the ROS level exceeds the defense mechanisms and is able to threaten cells by causing macromolecule damage and ultimately leads to cell death^5^. The size, surface area, and action of nanomaterials result in a high level of ROS that play a critical role in phytotoxic mechanisms^6^. Some researches on the environmental toxicity of NPs indicate the various potential mechanisms in which ZnO-NPs have induced plant damage^7,8^. ZnO-NPs induce LOX activity and membrane or DNA dysfunctions according to small volumes, large surface area, and ROS production^9^. Furthermore, the content of malondialdehyde (MDA) has been gradually increased with increasing levels of ZnO-NPs in *Cicer arietinum* seedlings^10^ indicating the accumulation of MDA at high levels of cellular damage that may come out from ROS^11^.

Excess zinc accumulation, which reaches phytotoxic levels, induces physiological changes, and inhibits growth by causing membrane oxidation and reducing the intake of fundamental nutrients^12^. Excess Zn interferes with the root loading site and reduces the rate of translocation or absorption of essential plant nutrients or causes mineral imbalances^13^. Plants have generated defensive antioxidant systems to counteract the uncontrolled output of ROS^14^. Antioxidants, non-enzymatic and enzymatic forms, prevent or suppress ROS reactions and delay or prevent cell damage^15^. Studies have shown that redox-enzymes can save cells from the harmful actions of ROS^14,16,17^. The phenylpropanoid biosynthesis pathway is stimulated under (a)biotic stressors that lead to the accumulation of various phenols that have the ability to break down harmful ROS^18^. In plants, heavy metals activate the phenylpropanoid pathway by regulating the biosynthesis of key enzymes such as PAL^19^. Polyphenol oxidase also helps break down ROS and increases plant tolerance to abiotic stresses^19,20^. The mechanism of action of the redox-enzyme may rely on the concentrations of nanoparticles, plant species, nanoparticle types, and ecosystems^21^.

*Punica granatum* L. (pomegranate) is an old fruit tree, known as a “Super-food”, which has an extended history of medicinal uses as a herbal remedy for cancer, diarrhea, diabetes, blood pressure, leprosy, dysentery, bleeding, bronchitis, indigestion, and infections^22^. There are more than 1000 varieties, originating from the Middle East^23^, in Egypt, about 8080 hectares planted^24^ and an agricultural policy aimed at increasing production by expansion in reclaimed areas.

Despite numerous publications on plant tolerance mechanisms and antioxidant functions in plants exposed to heavy metals, woody plants have not been adequately studied in this area^25^. Moreover, the kinetics of the enzyme is important for assessing the function of the enzyme in the metabolic process. The tests aimed at enriching knowledge about ZnO-NPs interactions with activity, the kinetics of redox-enzymes, and essential nutrients. Studies of pomegranate plants (woody plant) were performed in vitro conditions on callus tissue grown on MS medium with ZnO-NPs compared to salt using ZnO-BPs on redox-enzymes and essential minerals.

## Materials and methods

### ZnO-NPs and ZnO-BPs solutions

ZnO-NPs (99.5% purity; particle size less than 100 nm and surface area 15–25 m^2^g^−1^) were purchased from Sigma-Aldrich Company, St. Louis, MO, USA. ZnO-BPs used to be 99.999% mineral powder. ZnO-NPs and ZnO-BPs were individually dissolved directly in distilled H_2_O and dispersed using ultrasonic vibration (100 W, 40 kHz).

### Tissue culture and treatments

Murashige & Skoog media (MS) is composed of 4.4 g L^−1^ MS, 3% sucrose, 6 mg 2,4-D, 1 mg AsA, 5 mg adenine, 5 mg casein, 10 mg potassium dihydrogen phosphate, and different concentrations (0, 10, and 150 µg mL^−1^) of pre-prepared ZnO-NPs or BPs^26^. The pH was adjusted to 5.7, then, 3 g L^−1^ gel-rite was added, sterilized at 121 °C and a pressure of 105 kPa.

Fresh and healthy leaves were taken from pomegranate trees (*Punica granatum* cultivar Hegazy), washed under running tap water, and sterilized with 30% NaClO^27^. The sterilized-leaves were soaked in 150 mg L^−1^ AsA and 100 mg citric acid for 20 min and washed with sterilized-water^28^. In a 195 mL jar, three sterile leaf pieces (1–1.5 cm) were transplanted into 30 mL of sterilized and solidified MS medium. These culture media were transferred to a growth chamber (16/8 h, photoperiod 30 µM m^−2^ S^−1^ irradiance, temperature 25 ± 1 °C, relative humidity 50–60%)^4^. Some calli (4 weeks) were frozen in liquid nitrogen and stored at − 80 °C for enzyme analysis, and the anther were dried at 60 °C for 48 h for mineral identification. Twenty-five jars were applied in each treatment.

### Enzyme extraction

The frozen callus (0.5 g) was ground to a fine powder in liquid nitrogen and homogenized in 5 mL of 100 mM potassium phosphate buffer (PPB; pH 7.8) containing 0.1 mM EDTA (ethylenediaminetetraacetic acid) and 0.1 g PVP (polyvinylpyrrolidone). The supernatants were used to test the activity and kinetics of the examined enzymes after centrifugation of the mixture (4 °C, 18,000 rpm, 10 min). The protein content in the extract was estimated^29^.

### Lipoxygenase (EC 1.13.11.12)

LOX activity was tested following the technique of Minguez-Mosquera et al.^30^. To 5 mL of distilled H_2_O, 5 µL of Tween-20 and 35 µL of linoleic acid were combined and the pH was adjusted to 9.0 using 200 mM NaOH until the mixture was clear. After the pH was adjusted to 6.5 by HCl, the total volume was adjusted to 100 mL by 100 mM PPB. Absorption was tested after 50 µL of enzyme aliquots were incorporated into the reaction medium (2.95 mL). The alteration in absorbance per unit protein within 1 min (DA_234_ mg protein^−1^ min^−1^) was measured as an activity.

### Superoxide dismutase (EC 1.15.1.1)

SOD activity was tested after epinephrine oxidation^31^. The substrate mixture (3 mL) included Na_2_CO_3_ buffer (0.05 M; pH 10.2), 0.1 mL EDTA, 0.05 mL enzyme aliquot and 100 μL epinephrine. The alteration in absorption per unit protein within 1 min was measured at 480 nm (DA_480_ mg protein^−1^ min^−1^) as an activity.

### Catalase (EC 1.11.1.6)

CAT activity was examined one minute after H_2_O_2_ consumption^32^. The reaction method included 50 mM PPB (pH 7), 100 μL H_2_O_2_ (10 mM), and a 20 μL enzyme aliquot. Absorbance was examined and the difference in absorbance per unit protein within 1 min (DA_240_ mg protein^−1^ min^−1^) was measured as an activity.

### Peroxidase (EC 1.11.1.7)

POD activity was examined after tetraguaiacol formation^33^. The assay medium included PPB (30 mM, pH 7), hydrogen peroxide (6.5 mM), guaiacol (1.5 mM), and an enzyme aliquot (100 µL). Absorbance was examined and the difference in absorbance per unit protein within 1 min (DA_470_ mg protein^−1^ min^−1^) was measured as an activity.

### Ascorbate peroxidase (EC 1.11.1.11)

The activity of APX was examined after AsA oxidation^34^. The reaction medium contained PPB pH 7.0 (50 mM), ETDA (0.1 mM), hydrogen peroxide (1.2 mM), AsA (0.5 mM), and an enzyme aliquot (50 µL). The absorbance was examined and the change in the absorbance per unit protein within 1 min (DA_290_ mg protein^−1^ min^−1^) was measured as an activity.

### Phenylalanine ammonia-lyase (EC 4.3.1.5)

PAL activity was bio-assayed after trans-cinemate production^35^. The experiment medium (2 mL) consisted of 50 mM borate buffer (pH 8.7), 1 mg L^−1^ phenylalanine, 0.5 mL enzyme aliquot, and was left for one hour at 37 °C. The absorbance was checked after the termination of the reaction with 1 mL of HCl (0.5 N) and centrifugation (5 min, 2000 rpm). The change in absorption per unit protein within 1 min (DA_290_ mg protein^−1^ min^−1^) was measured as an activity.

### Polyphenol oxidase (EC 1.14.18.1)

The activity of PPO was tested after the production of purpurogallin^36^. The mixture medium (2 mL) consisted of 100 mM PPB (pH 6), 1 mL catechol (100 mM), enzyme aliquot (200 µL), and was left at 25 °C (5 min). The absorbance was monitored at 495 nm after the termination of the reaction with 1 mL of H_2_SO_4_ (2.5 N). The alteration in absorbance per unit protein within 1 min (DA_495_ mg protein^−1^ min^−1^) was measured as an activity.

### Kinetic parameters

The kinetic activities of all tested enzymes were studied using different levels of enzyme aliquot and data plotted as 1/V and 1/S^37^. Michaelis constant (K_m_), maximum velocity (V_max_), catalytic rate constant (K_cat_) values were evaluated by Michaelis–Menten plots.

### Minerals

Dry callus tissues were digested by perchloric acid (60%), nitric acid (Analar), and sulfuric acid (Analar) in a 1: 3: 1 ratio for the assay copper, iron, potassium, magnesium, manganese, phosphorous, and zinc^4^. Potassium and P were assayed in the digested samples^4^ and were expressed as mg g^−1^ dry weight (DW). Other minerals (Cu, Fe, Mg, Mn, and Zn) were measured using atomic absorption spectrophotometry (Buck's 210 Vgp model, USA) and were expressed as mg g^−1^ DW.

### Statistical analysis

Present values (± standard deviation) have been averages of four biological replicates, with all three technical replicates, in most cases, and analysis was performed by SPSS software (version 22). One-way test (ANOVA) and Tukey's test were performed for multiple comparisons (*P* ≤ 0.05). Correlation tests (Pearson correlation) were carried to achieve the relation between the mean rate of various criteria of pomegranate under ZnO-NPs and BPs. Asterisks indicate a significant correlation (* and ** at 5 and 1%, respectively). Correspondence analysis was used to analyze relationships between redox-enzyme activities or mineral concentrations and different concentrations of ZnO particles.

## Results

### Lipoxygenase

**Figure 1:**
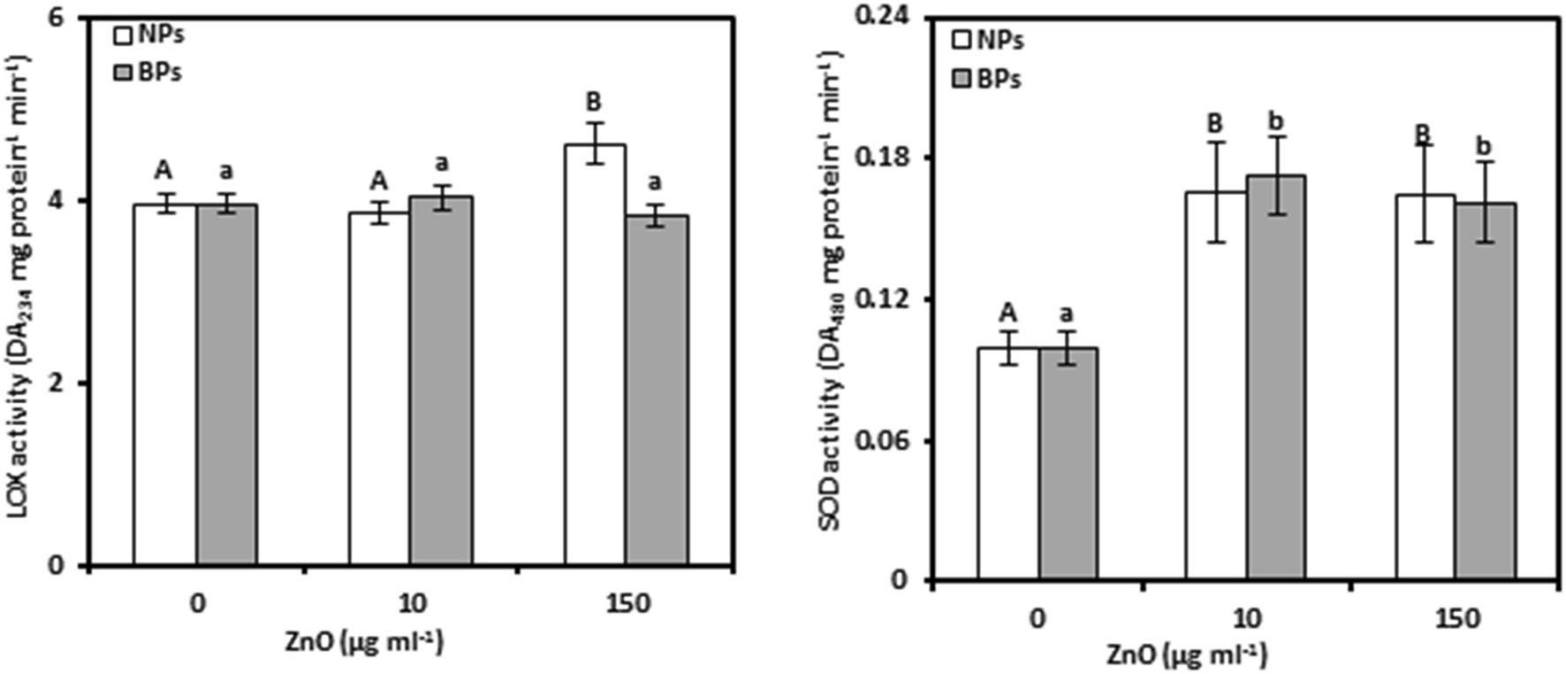
Lipoxygenase (LOX; a) and superoxide dismutase (SOD; b) activity of *Punica granatum* calli under the influence of different concentrations of ZnO-NPs and BPs for 28 days. Data are means ± SD (n = 4). The different letters, capital for NPs treatments and small for BPs, indicate statistically significant differences (*P* ≤ 0.05).

The activities of LOXs were assessed in pomegranate calli exposed to different levels of ZnO-NPs or BPs to consider the degree of membrane damage (Fig. 1a, Tables S1, 2). The data showed that ZnO-NPs at the low level failed to significantly enhance LOX activity, while the high level promoted such activity by about 17%, compared to the controls. However, the different levels of ZnO-BPs did not considerably alter LOX activity. ZnO-NPs showed a significant correlation between LOX activity (0.857**) and Zn content, whereas in the case of BPs this relationship was insignificant (− 0.552).

### Superoxide dismutase

SOD activity was assessed as an important scavenger for ROS (Fig. 1b, Tables S1, and S2). In pomegranate calli, SOD activity was induced by increased levels of ZnO-NPs or BPs in the nutrient medium. Compared to untreated controls at treatments of 10 and 150 μg mL^−1^, the elevation was 66.45 and 65.31% in NPs and 72.98 and 61.71% in BPs, respectively. Moreover, in the case of NPs, the correlation between SOD activity and Zn concentration was significant (0.710*), while it was insignificant (0.448) in the BPs.

### Catalase

**Figure 2:**
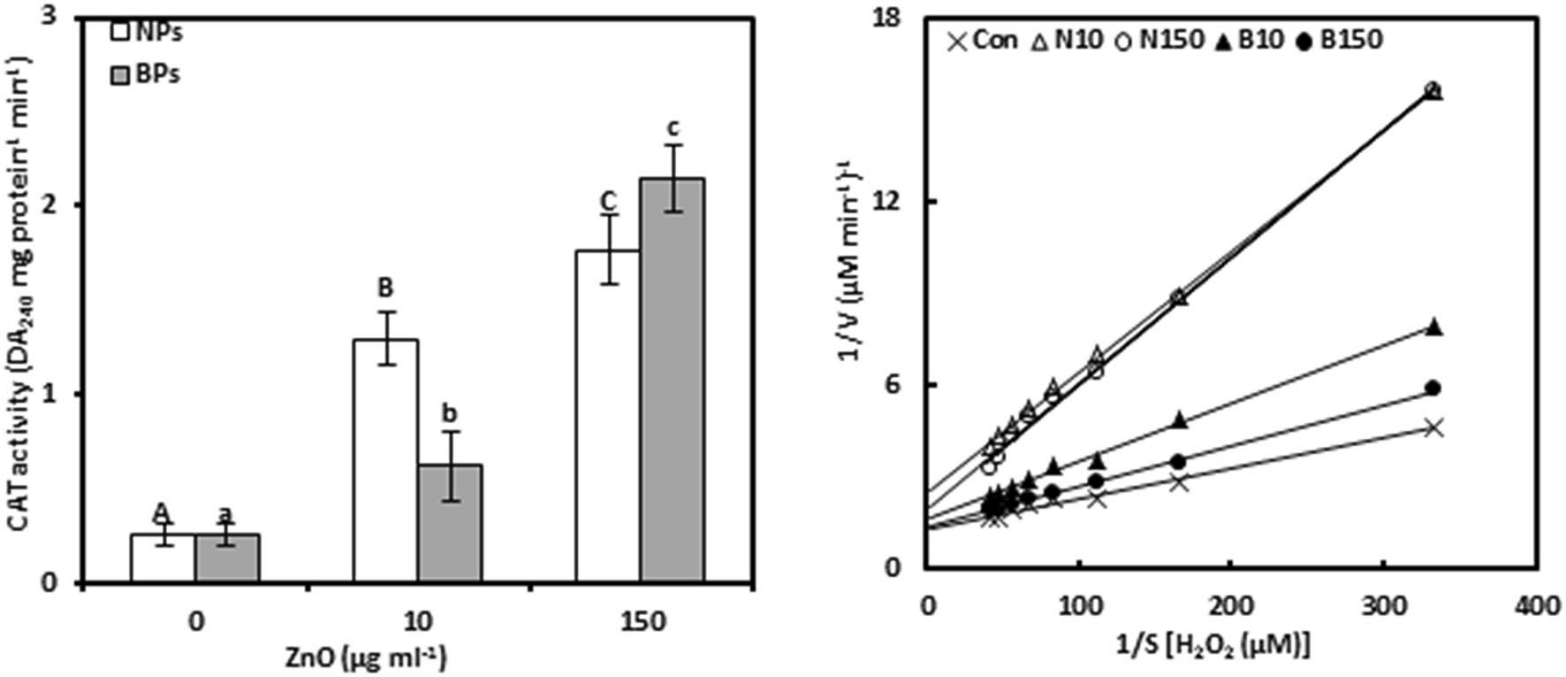
Catalase activity (CAT; a) and Lineweaver–Burk curve of CAT enzyme (b) of *Punica granatum* calli under the influence of different concentrations of ZnO-NPs and BPs for 28 days. Data are means ± SD (n = 4). The different letters, capital for NPs treatments and small for BPs, indicate statistically significant differences (*P* ≤ 0.05).

CAT activity was evaluated in pomegranate calli exposed to different levels of NPs or BPs because it helps in dismutation H_2_O_2_ into H_2_O and O_2_ (Fig. 2a, Tables S1, 2). CAT activity was stimulated with increased levels of ZnO-NPs or BPs, with the highest concentration of ZnO-BPs (150 µg mL^−1^) showing an 8.34-fold higher increase in CAT activity compared to ZnO-NPs which resulted in 6.87-fold over the non-treated-control. Additionally, the result showed that CAT activity in the case of NPs (0.835**) and BPs (0.989**) represented significant correlations with zinc contents in callus tissues.

Furthermore, various assays were performed to test kinetic activity with respect to the CAT enzyme under different concentrations of ZnO-NPs or ZnO-BPs (Fig. 2b, Table 1, Tables S3, 4). An increase in NPs or BPs treatments led to a catalytic effect on K_m_ of CAT. For example, in the low and high treatments of NPs or BPs, the K_m_ increased to 105.93, 246.59, 50.54, and 30.19%, respectively; higher than the control. Additionally, the NPs showed a significant correlation between the K_m_ and the Zn content (0.963**), however, this relationship in the case of BPs was minimal (0.245).

**Table 1:** Michael constant (K_m_), maximum activity (V_max_), and catalytic (K_cat_) of catalase (CAT), peroxidase (POD), ascorbate peroxidase (APX), phenylalanine ammonia-lyase (PAL), and polyphenol oxidase (PPO) enzymes of *Punica granatum* calli under the influence of different concentrations of ZnO-NPs and BPs for 28 days.

Enzyme	Treatments (μg mL^−1^)		K_m_ (μM)	V_max_ (μmol min^−1^)	K_cat_ (min^−1^)
CAT	0	0	0.0076 ± 0.0001Cc	0.7512 ± 0.0084Aa	0.0276 ± 0.0003Bb
	NPs	10	0.0157 ± 0.0002B	0.4011 ± 0.0094C	0.0260 ± 0.0006B
		150	0.0264 ± 0.0004A	0.6330 ± 0.0262B	0.0377 ± 0.0015A
	BPs	10	0.0115 ± 0.0003a	0.6074 ± 0.0065b	0.0357 ± 0.0003a
		150	0.0099 ± 0.0003b	0.7293 ± 0.0193a	0.0367 ± 0.0009a
POD	0	0	0.1052 ± 0.0008Ab	0.2391 ± 0.0021Aa	0.0088 ± 0.0001Cb
	NPs	10	0.0388 ± 0.0005C	0.1595 ± 0.0011C	0.0103 ± 0.0001B
		150	0.0630 ± 0.0006B	0.2006 ± 0.0010B	0.0120 ± 0.0001A
	BPs	10	0.1086 ± 0.0002a	0.1412 ± 0.0011c	0.0080 ± 0.0001c
		150	0.0782 ± 0.0016c	0.2146 ± 0.0025b	0.0108 ± 0.0001a
APX		0	0.4328 ± 0.0026Bc	1.3014 ± 0.0796Ab	0.0478 ± 0.0029Bc
	NPs	10	0.4290 ± 0.0015B	0.8640 ± 0.00197C	0.0560 ± 0.0001A
		150	0.4415 ± 0.0029A	0.9420 ± 0.0036B	0.0562 ± 0.0002A
	BPs	10	0.8293 ± 0.0035b	1.2333 ± 0.0048b	0.0726 ± 0.0002b
		150	1.3792 ± 0.0066a	1.9756 ± 0.0141a	0.0995 ± 0.0007a
PAL		0	0.0131 ± 0.0002Aa	0.2546 ± 0.0036Ac	0.0094 ± 0.0001Cc
	NPs	10	0.0056 ± 0.0001C	0.2326 ± 0.0012B	0.0151 ± 0.0001A
		150	0.0066 ± 0.0001B	0.2236 ± 0.0031C	0.0133 ± 0.0001B
	BPs	10	0.0047 ± 0.0001b	0.3086 ± 0.0007b	0.0182 ± 0.0001b
		150	0.0026 ± 0.0001c	0.3783 ± 0.0013a	0.0191 ± 0.0001a
PPO		0	0.3464 ± 0.0049Aa	0.3297 ± 0.0048Aa	0.0121 ± 0.0001Aa
	NPs	10	0.1384 ± 0.0008C	0.0634 ± 0.0009C	0.0041 ± 0.0001C
		150	0.1754 ± 0.0007B	0.1248 ± 0.0004B	0.0074 ± 0.0001B
	BPs	10	0.3098 ± 0.0024c	0.1679 ± 0.0064c	0.0099 ± 0.0003c
		150	0.3298 ± 0.0046b	0.2249 ± 0.0026b	0.0113 ± 0.0001b

Data are means ± SD (n = 4). The different letters, capital for NPs treatments and small for BPs, indicate statistically significant differences (*P ≤ *0.05).

NPs or BPs treatments reduced V_max_ of the CAT enzyme by 46.61, 15.74, 19.15 and 2.93%, respectively, compared to the control. However, the correlation between V_max_ and Zn in the NPs or BPs treated callus was insignificant.

The K_cat_ rate of CAT was increased in NPs or BPs-treated calli, only 10 µg mL^−1^ NPs decreased it slightly. The high level of NPs or BPs (150 µg mL^−1^) stimulated K_cat_ by 36.73 and 33.06%, respectively, over the controls. Moreover, NPs and BPs treatments showed a marked positive association between K_cat_ and Zn content (0.948** and 0.711*, respectively).

### Peroxidase

**Figure 3:**
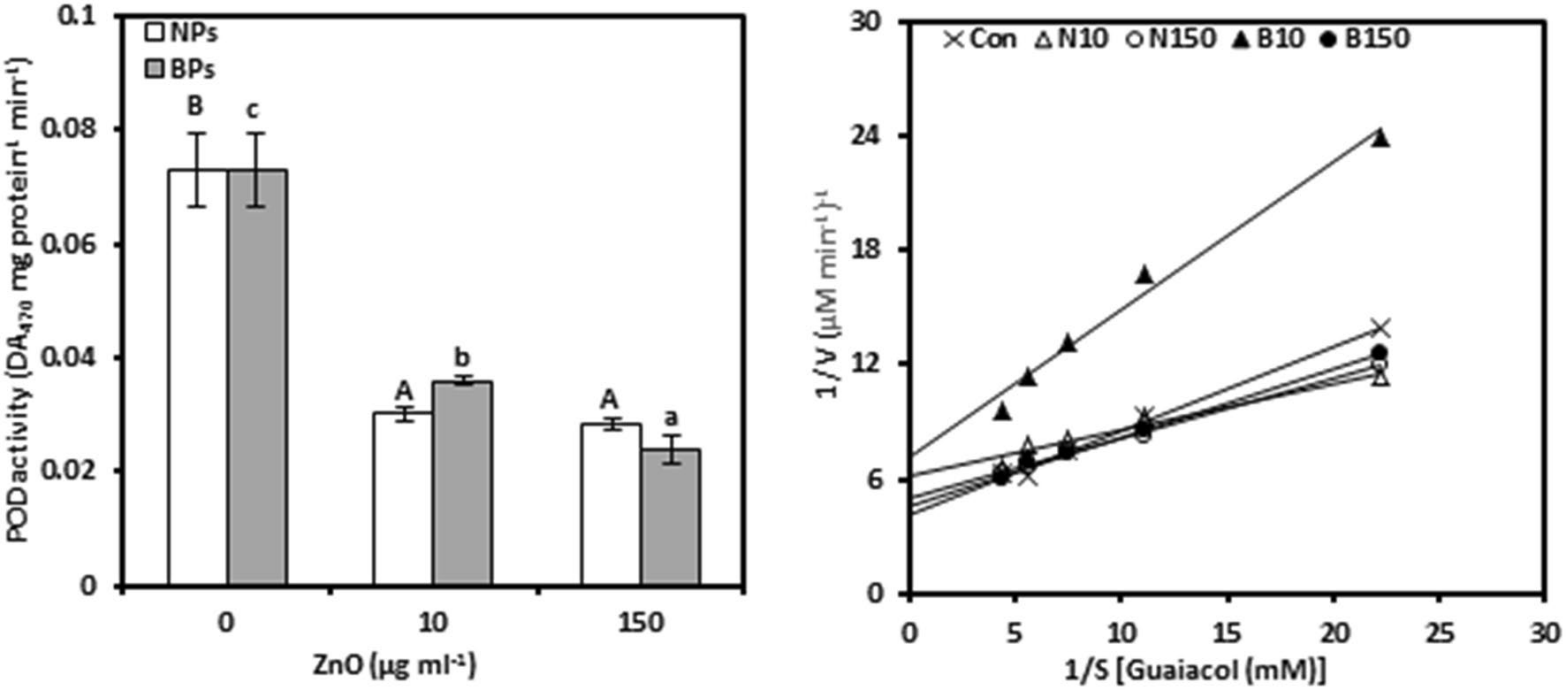
Peroxidase activity (POD; a) and Lineweaver–Burk curve of POD enzyme (b) of *Punica granatum* calli under the influence of different concentrations of ZnO-NPs and BPs for 28 days. Data are means ± SD (n = 4). The different letters, capital for NPs treatments and small for BPs, indicate statistically significant differences (*P* ≤ 0.05).

POD activity was evaluated as it stimulates the oxidation by H_2_O_2_ of a broad range of organic (Fig. 3a, Tables S1, 2). Treatments of pomegranate calli with ZnO-NPs or BPs negatively affected POD activity. The decrease in activity at 150 µg mL^−1^ of ZnO-NPs or BPs treatments, compared to the controls, was 61.07 and 67.12%, respectively. As expected, ZnO-NPs or BPs treatments showed negative relationships between POD activity and Zn concentrations (− 0.663 and − 0.794*, respectively).

For POD kinetic activity, ZnO-NPs or BPs treatments reduced the K_m_ and V_max_, with K_m_ only slightly stimulated by 10 μg mL^−1^ BPs (Fig. 3b, Table 1, Tables S5^6^). Compared with control calli, 10 µg mL^−1^ treatments of ZnO-NPs or ZnO-BPs showed a higher decrease in K_m_ and V_max,_ compared to 150 µg mL^−1^, which caused reductions of 40.10%, 25.73%, 16.09%, and 10.23%, respectively. In other words, the increased concentration of NPs or BPs caused a stimulating effect on K_cat_, only 10 µg mL^−1^ BPs significantly reduced it. The maximum increase in K_cat_ was observed at 36.15 and 23.06%, respectively, at 150 μg mL^−1^ ZnO-NPs and BPs.

### Ascorbate peroxidase

**Figure 4:**
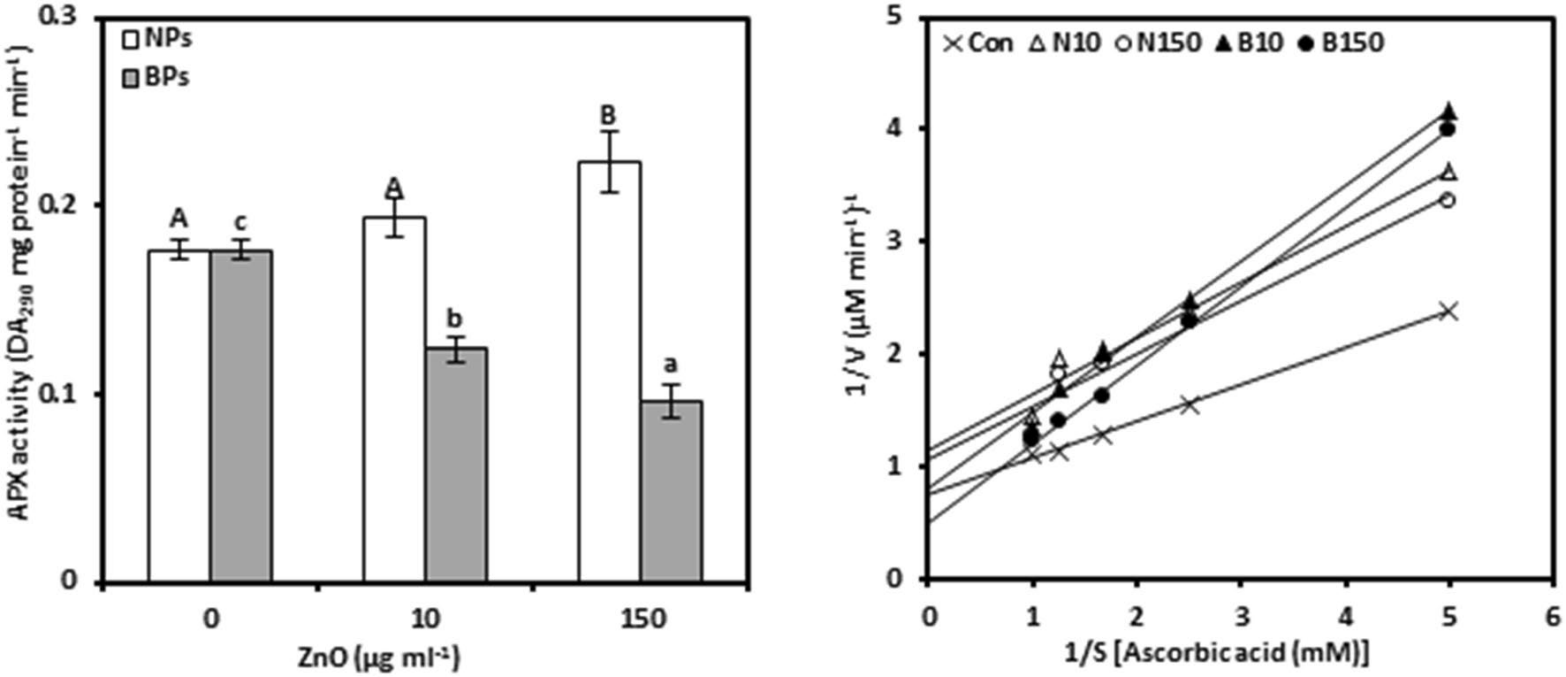
Ascorbate peroxidase activity (APX; a) and Lineweaver–Burk curve of APX enzyme (b) of *Punica granatum* calli under the influence of different concentrations of ZnO-NPs and BPs for 28 days. Data are means ± SD (n = 4). The different letters, capital for NPs treatments and small for BPs, indicate statistically significant differences (*P* ≤ 0.05).

APX activity was evaluated as it catalyzes the hydrogen peroxide dependent oxidation of ascorbate in plants (Fig. 4a, Tables S1 and S2). Increasing ZnO-NPs levels from 10 to 150 μg mL^−1^, increased APX activity in pomegranate callus tissues, showing only 9.77 and 26.79% increases over untreated calli, respectively. However, ZnO-BPs inhibited the activity as indicated by 29.93 and 45.73% at 10 and 150 µg mL^−1^, respectively, relative to the controls. Moreover, under NPs treatments, APX activity showed a strong positive association with zinc concentration (0.868**), while the same relationship was negatively strong in the case of BPs (− 0.826**).

APX kinetic activity was affected differently by increasing the level of ZnO-NPs or BPs in the nutrient medium (Fig. 4b, Table 1, Tables S7 and S8). The data revealed that no considerable changes were observed in the K_m_ of APX enzyme in calli treated with ZnO-NPs. Moreover, NPs treatments reduced the V_max_ of APX enzyme by 33.61% and 27.61% at 10 and 150 µg mL^−1^ respectively, compared to the controls. However, increased concentrations of NPs stimulated the K_cat_ of APX enzyme as shown by 17.09% and 17.46% at 10 and 150 µg mL^−1^, respectively, over the control. Regarding ZnO-BPs, most APX kinetic parameters were increased with increasing concentration of BPs, only V_max_ slightly decreased at 10 µg mL^−1^.

### Phenylalanine ammonia-lyase

**Figure 5:**
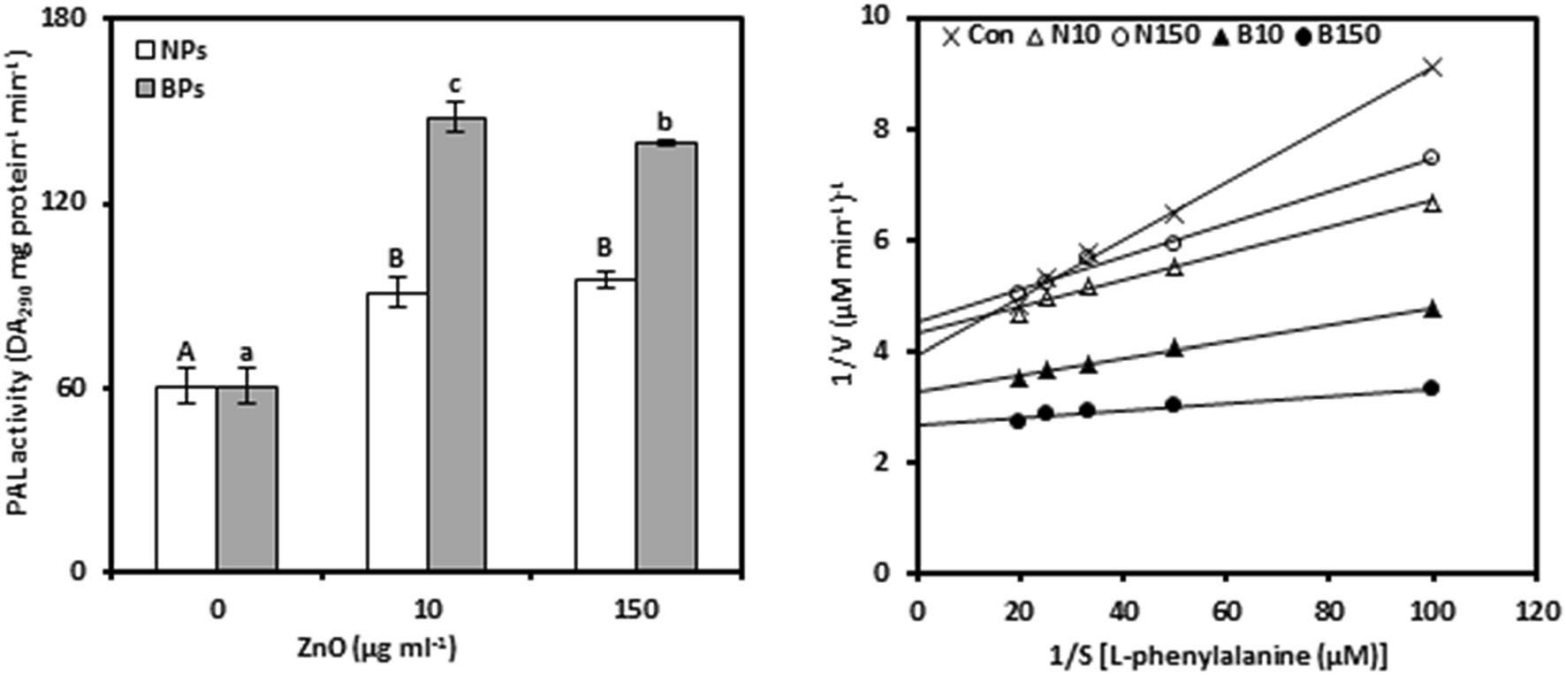
Phenylalanine ammonia-lyase activity (PAL; a) and Lineweaver–Burk curve of PAL enzyme (b) of *Punica granatum* calli under the influence of different concentrations of ZnO-NPs and BPs for 28 days. Data are means ± SD (n = 4). The different letters, capital for NPs treatments and small for BPs, indicate statistically significant differences (*P* ≤ 0.05).

PAL was analyzed to mark whether ZnO-NPs or ZnO-BPs treatments affected phenolic biosynthesis in pomegranate calli (Fig. 5a, Tables S1 and S2). PAL activity, compared to the controls, was significantly increased in response to the concentrations of NPs (10 and 150 µg mL^−1^) by 49.82% and 56.95%, respectively. A further increase in PAL activity was observed with increasing concentrations of BPs by 144.20% and 130.09%, respectively, at 10 and 150 µg mL^−1^, above the controls. In addition, our results revealed that PAL activity under NPs (0.631) and BPs (0.573) represented an insignificant relationship with Zn content in callus tissues.

Compared to the control, treatments with ZnO-NPs reduced K_m_ and V_max_ as indicated by a decrease of 49.64 and 12.17% at 150 µg mL^−1^, respectively (Fig. 5b, Table 1, Tables S9, 10). Similarly, a decrease in the K_m_ of PAL enzyme was found in the ZnO-BPs-treated callus; however, stimulation was observed in V_max_. In contrast, treatments using ZnO-NPs or BPs significantly increased the K_cat_ of PAL enzyme. BPs treatments showed a higher increase in K_cat_ of 94.15 and 103.69% of NPs resulting in increases of 61.14 and 42.51% at 10 and 150 μg mL^−1^, respectively, over the controls.

### Polyphenol oxidases

**Figure 6:**
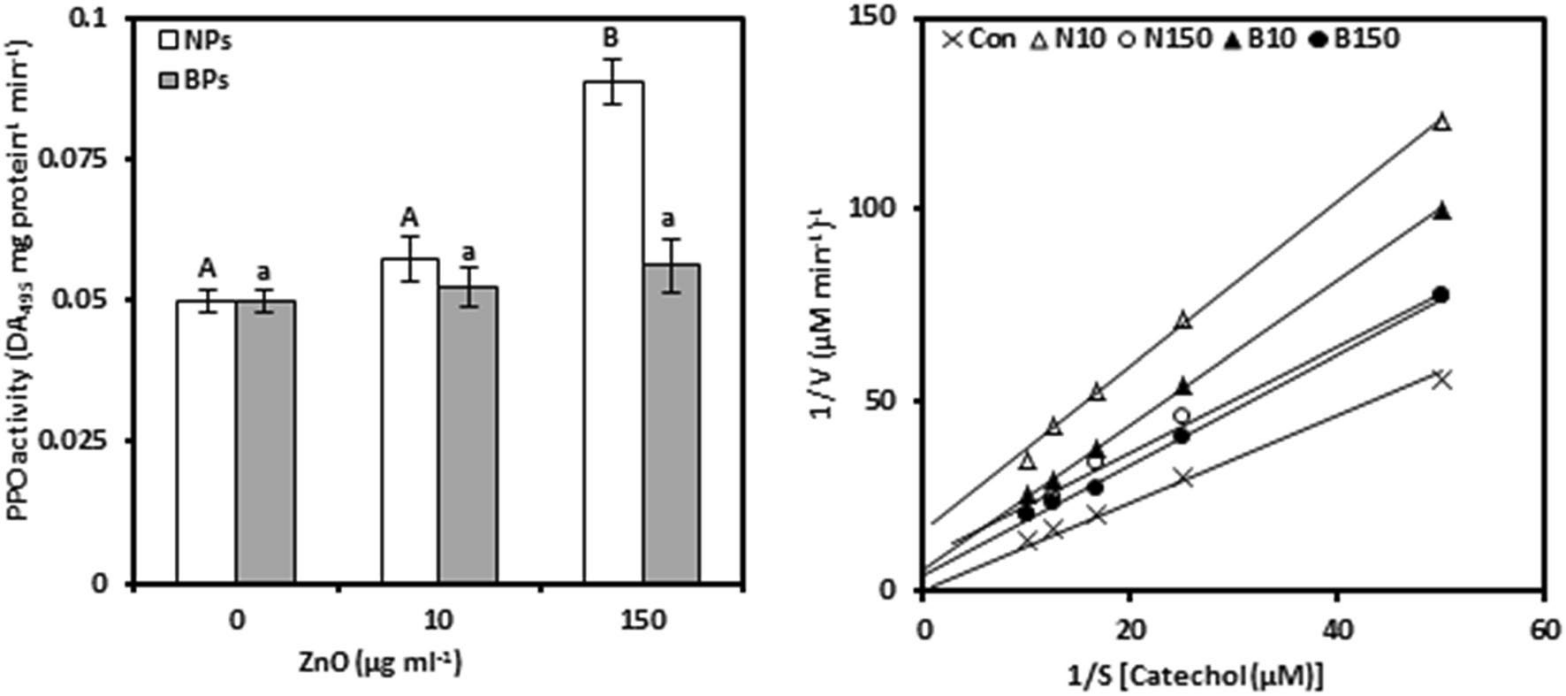
Polyphenol oxidase activity (PPO; a) and Lineweaver–Burk curve of PPO enzyme (b) of *Punica granatum* calli under the influence of different concentrations of ZnO-NPs and BPs for 28 days. Data are means ± SD (n = 4). The different letters, capital for NPs treatments and small for BPs, indicate statistically significant differences (*P* ≤ 0.05).

PPO activity was tested in pomegranate calli to consider at the degree of oxidation of phenolic compounds induced by ZnO-NPs or ZnO-BPs treatments (Fig. 6a, Tables S1, 2). PPO activity showed insignificant change in response to 10 µg mL^−1^ ZnO-NPs; however, it was 77.97% improved over the control value when exposed to 150 µg mL^−1^ ZnO-NPs. Otherwise, no significant changes were observed in PPO activity by applying BPs to pomegranate calli. Moreover, the result showed strong positive correlations between PPO activity and Zn concentrations in the ZnO-NPs- or BPs-treated callus (0.979** and 0.949**, respectively).

The influence of NPs or BPs treatments on PPO kinetic activity of pomegranate calli was also studied (Fig. 6b, Table 1, Tables S11 and S12). All of the investigated kinetic parameters were reduced concerning PPO enzyme whatever of the concentration of NPs or BPs. Compared to the control, the highest reduction in K_m_, V_max_, and K_cat_ of PPO enzyme was found in ZnO-NPs treatments to be 60.06%, 80.76%, and 66.07% at 10 µg mL^−1^, and 49.36%, 62.16%, and 38.60% at 150 µg mL^−1^, respectively. Likewise, exposure to BPs resulted in a statistically slight decrease in K_m_, V_max_, and K_cat_ and the decrease, relative to the control, was 10.57%, 49.07%, and 18.44% at 10 µg mL^−1^, and 4.79%, 31.77%, and 6.48% at 150 µg mL^−1^, respectively.

### Minerals

To determine whether ZnO-NPs or ZnO-BPs treatments affect the uptake of the macro-elements, K, Mg, and P, these elements were analyzed in the tissues of pomegranate callus (Table 2, Tables S1, 2). Relative to the control, the different concentrations concerning NPs or BPs showed insignificant changes in the accumulation of K and P in callus tissues. Moreover, the data revealed non-significant correlations between K, P, and Zn concentrations in calli treated with ZnO-NPs or BPs, only significant between P and Zn (0.766*) under BPs conditions. The accumulation of Mg reduced with an increase in the level of NPs or BPs within the nutrient medium. Compared to the control, the largest decrease of 41.50% and 39.16%, respectively, was observed in Mg accumulation at a concentration of 150 µg mL^−1^ ZnO-NPs or BPs. Moreover, ZnO-NPS or BPs treatments showed strong negative correlations between the Mg and Zn concentrations (− 0.737* and − 0.890**, respectively).

**Table 2:** Mineral concentrations of *Punica granatum* calli under the influence of different concentrations of ZnO-NPs and BPs for 28 days.

Treatments (μg mL^−1^)		Zinc	Manganese	Iron	Copper	Potassium	Magnesium	Phosphorus
	0	1.786 ± 0.055C	0.540 ± 0.030C	25.560 ± 0.362A	1.300 ± 0.170A	8.098 ± 0.620A	4.507 ± 0.021A	205.864 ± 23.04A
NPs	10	2.677 ± 0.025B	0.902 ± 0.017B	14.515 ± 0.100B	0.452 ± 0.033B	6.883 ± 0.748A	2.917 ± 0.096B	240.076 ± 20.86A
	150	6.772 ± 0.005A	1.300 ± 0.018A	7.624 ± 0.103C	0.251 ± 0.013B	6.852 ± 0.717A	2.637 ± 0.044C	242.646 ± 6.68A
	0	1.786 ± 0.055c	0.540 ± 0.030c	25.560 ± 0.362a	1.300 ± 0.170a	8.098 ± 0.620a	4.507 ± 0.021a	205.864 ± 23.04a
BPs	10	2.422 ± 0.036b	0.879 ± 0.057b	24.138 ± 1.029a	0.599 ± 0.010b	6.952 ± 0.492a	3.412 ± 0.131b	212.934 ± 6.44a
	150	4.849 ± 0.007a	1.286 ± 0.079a	15.260 ± 0.013b	0.421 ± 0.011b	6.834 ± 0.423a	2.742 ± 0.001c	243.944 ± 17.24a

Data are means ± SD (n = 4). The different letters, capital for NPs treatments and small for BPs, indicate statistically significant differences (P ≤ 0.05).

In addition, the effect of NPs and BPs treatments on micronutrient accumulation in pomegranate callus was studied (Table 2, Tables S1, 2). Copper concentrations gradually reduced with increasing ZnO-NPs or BPs in the nutrient medium (relative to the control, 65.21%, and 80.70% decreased for NPs, 53.92%, and 67.58% for BPs at 10 and 150 µg mL^−1^, respectively). Similarly, Fe concentration significantly decreased in callus tissues exposed to ZnO-NPs (43.21% and 70.17%, at 10 and 150 µg mL^−1^, respectively), while its concentration only decreased at 150 µg mL^−1^ ZnO-BPs (40.30%). In contrast, Mn concentration increased with increasing levels of NPs or BPs in the nutrient medium as evidenced by 66.86%, 62.68%, 140.61%, and 138.00% at 10 and 150 µg mL^−1^ respectively, over the control. Further, the results showed that the Cu and Fe concentrations represented a strong negative association with the Zn concentration in NPs- or BPs-treated calli (− 0.754*, − 0.772*, − 0.883**, and − 0.992**, respectively), whereas, the correlation was positive between Mn and Zn contents (0.945** and 0.951**, respectively).

The uptake of zinc into the callus was dependent on the concentration and size of the particles (Table 2). As expected, the gradual increase in ZnO-NPs or BPs treatments increased zinc accumulation in the callus tissues. The largest increase in Zn concentration was observed to be 279.23% within callus tissues exposed to 150 µg mL^−1^ NPs, compared to BPs (171.52%), over the control.

### Correspondence analysis

**Figure 7:**
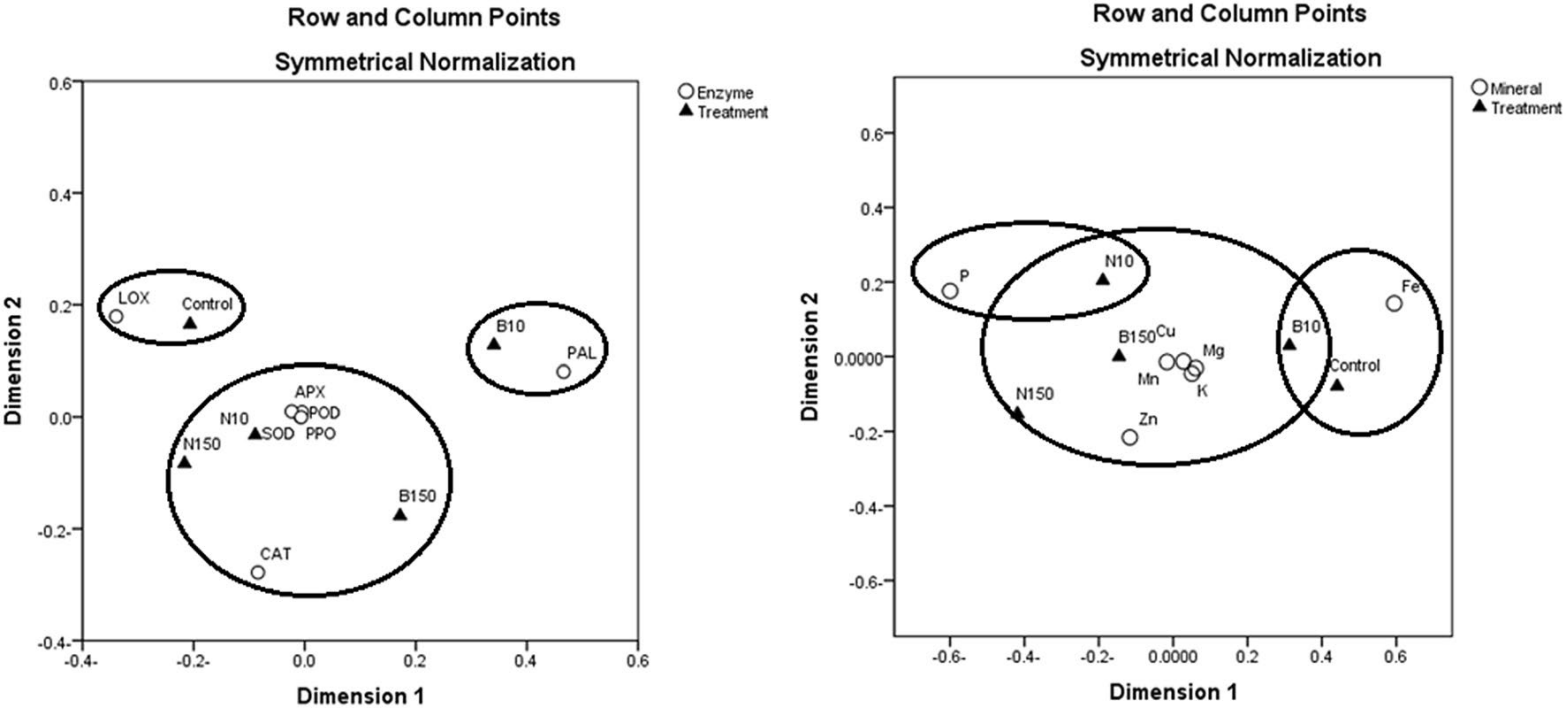
Correspondence analysis of redox-enzyme activities (**a**) and mineral concentrations (**b**) of *Punica granatum* calli under the influence of different concentrations of ZnO-NPs and BPs for 28 days. N10 and N150 = ZnO-NPs 10 and 150 μg mL^-1^; B10 and B150 = ZnO-BPs 10 and 150 μg mL^-1^; LOX = lipoxygenase; SOD = superoxide dismutase; CAT = catalase; POD = peroxidase; APX = ascorbate peroxidase; PAL = phenylalanine ammonia-lyase; PPO = polyphenol oxidase.

Correspondence analysis confirmed strong correlations between the activities of CAT, SOD, POD, APX, PPO, and both levels of ZnO-NPs and the high level of ZnO-BPs (Fig. 7a,b). Moreover, it showed a close association between K, Mg, Cu, Mn, Zn, ZnO-NPs, and BPs.

## Discussion

Lipid decomposition and peroxidation of membrane-bound fatty acids under stress conditions are associated with elevated LOX activity^38^. Our results revealed that the high level of NPs boosted LOX activity, while the low level (10 µg mL^−1^), and both levels of BP failed to significantly boost its activity in the calli. These results indicate that callus tissues, exposed to the low level of ZnO-NPs and both levels of ZnO-BPs, may contain sufficient endogenous antioxidants to remove the low ROS content. Strong correlations between LOX activity and zinc concentration confirmed a previous review that ZnO-NPs increase O_2_^⋅−^ formation, leading to oxidative stress^39^. When ZnO-NPs reach the mitochondria, they induce ROS by interfering with their reactions that lead to the depolarization of mitochondrial membranes^40^. The insignificant effects of these concentrations of NPs and BPs indicate that these treatments may not have highly toxic effects and that the antioxidant systems were able to detoxify ROS.

Several enzymatic antioxidants have been increased under ZnO-NPs or BPs, indicating that these enzymes enable plants to withstand ZnO stress. SOD is a metallic enzyme^41^ that forms the primary line of protection against ROS; depending on the metal group, it is categorized into three main isozymes: Fe-SOD (found in plastids), MnSOD (found in mitochondria matrix, peroxisomes, cell wall), and Cu/Zn-SOD (found in cytosol, peroxisomes, and plastids)^42^. Statistics showed that ZnO-NPs or BPs stimulated SOD and CAT activities in callus tissues, indicating that ZnO catalyzed antioxidant enzymatic defense, which helped calli resisted this stress. Likewise, Zoufan et al.^43^ reported an increased SOD activity in leaves and roots of *Chenopodium murale* under the stress of ZnO-NPs. The SOD activity value, which decomposes O_2_^⋅−^ to H_2_O_2_, was changed to 1.7-fold higher in ZnO-NPs-treated calli, compared to 1.6-fold in 150 µg mL^−1^ ZnO-BPs. The elevated CAT activity, which degrades the H_2_O_2_ of the SOD product, in the callus tissues could indicate the callus tolerance of ZnO stress; as Stephenie et al.^44^ reported that elevated SOD activity, without the concomitant increase in H_2_O_2_ elimination capabilities, could lead to increased cytotoxicity. An increased SOD activity and Mn concentration with decreased Cu and Fe concentrations in callus tissues indicate an increased Mn-SOD isoenzyme activity. Likewise, the reduction in the available iron caused a shift to the use of the more available minerals, Mn, and as a result, Mn-SOD was second only to Fe-SOD^45^; the copper restriction also reduced the action of Cu/Zn-SOD^46^. Melchiorre et al.^47^ also found that higher Mn-SOD values were associated with tolerance to an abiotic stress in wheat. Our data displayed that CAT activity was significantly increased in ZnO-treated calli, indicating that exposure to ZnO-NPs or BPs increased ROS content in callus cells, but at the same time, they also catalyzed defense systems. A similar increase in CAT activity has been notified with the use of ZnO-NPs in mesquite plants^48^. Although the applications of NPs or BPs had a significant stimulatory effect on CAT activity, they reduced Fe accumulation in callus tissues. Santos et al.^49^ also declared that Fe deficiency increased CAT activity in inactive soybean strains. The strong correlation among CAT activity and Zn concentration may reveal that the CAT enzyme in calli is involved in antioxidant defense against ZnO-NPs or BP stress. However, it was unlikely that NPs-treated callus could successfully remove ROS due to its low ability to remove H_2_O_2_ and O_2_^⋅−^, resulting in a higher rate of the callus damage at high concentration (150 µg mL^−1^) compared to ZnO-BPs-treated callus. Consistent with these data, enhanced CAT activity was observed in mesquite seedlings^48^ and duckweed plants^50^ treated with ZnO-NPs. Strong positive correlations between CAT activity and manganese content in ZnO-NPs-treated calli (0.947**) or BPs (0.921**) confirm previous reports that the role of CAT in mitochondria is primarily correlated to the role of Mn-SOD in detoxification of mitochondria from ROS, which were generated during respiration of yeast^51^. Although the kinetic properties of redox-enzymes affected by heavy metals have been examined through numerous tests, the precise testing of the effects of ZnO-NPs is yet deficient. Current results showed that, in the presence of NPs or BPs in the nutrient medium, an increase, decrease, or no change in the K_m_, V_max_, and K_cat_ values of the redox-enzymes was obtained.

The K_m_ is an important kinetics parameter of a simple test rate for the affinity of a substrate with an enzyme that can be demonstrated^52^. A new observation is that the K_m_ (H_2_O_2_) of the CAT enzyme steadily increased as ZnO-NPs or ZnO-BPs increased. However, depending on the assumption that lower K_m_ values are a characteristic of enzyme function that was valid only at low substrate concentrations^53^, the differences between ZnO-NPs or ZnO-BPs may be associated with increased calli growth in ZnO-BPs. Rapid increases in the K_m_ (H_2_O_2_) of the CAT enzyme in ZnO-NPs or BPs-treated calli indicated that they are sensitive to ZnO. Our results showed that the CAT enzyme from callus treated with ZnO-BPs had a greater substrate affinity for H_2_O_2_ than that treated with ZnO-NPs. They also showed that the CAT from callus treated with BPs had a greater substrate affinity for H_2_O_2_ than that treated with NPs. The plot displayed a set of straight lines revealing that the V_max_ values were significantly decreased by ZnO-NPs treatments, but in the case of ZnO-BPs at 150 μg mL^−1^, the decrease was minimal. These results indicate that ZnO caused a mixed inhibition of the CAT enzyme, which indicate that ZnO could combine with the enzyme molecules additionally the enzyme–substrate-complex. The increase in K_cat_ of CAT was confirmed in previous reports by Malanoski et al.^54^ who concluded that the ZnO-NPs-enzyme complexes did not depend on the shifting of products to the surface of ZnO-NPs and the enzyme-complexes were directly influenced by ZnO-NPs engagement.

Current data showed that with increased levels of NPs or BPs in the nutrient medium, POD activity decreased in pomegranate callus tissues, and these findings are consistent with earlier work that found adverse impacts of ZnONPs treatments on POD activity in purslane roots^55^. Unlike CAT activity, POD activity considerably decreased in NPs or BPs treated calli. Inhibition of POD and stimulation of CAT activity confirm earlier reports that concluded that the balance of antioxidant systems in plants could be delayed or modified to revoke oxidative stress^56^. In addition, the obtained results indicated that CAT was negatively bound to Fe. However, the association between POD and Fe was positive under ZnO-NPs or BPs. This association may be related to the higher ability of CAT to detoxify H_2_O_2_ compared to POD under Fe deficiency, which resulted from the interfering of Zn with Fe uptake. Moreover, it can be suggested that the decreased POD activity was due to iron deficiency, which is essential for the biosynthesis of this enzyme molecule.

Another new observation was that the decrease in K_m_ and V_max_ of POD by ZnO-NPs treatments may be due to uncompetitive inhibition of ZnO-NPs. A similar effect was observed for ZnO-BPs at 150 µg mL^−1^ on K_m_, V_max_, and K_cat_ in POD. These results may indicate that ZnO may combine with an enzymatic substrate complex. Although the enzyme had a high affinity for the substrates and the K_cat_ was increased, the activity of this enzyme was low, and this may mean that the product interacts with ZnO. However, ZnO-BPs at the low level resulted in a corresponding increase in K_m_ and a decrease in V_max_ and K_cat_ of POD, which may indicate that BPs at the low level had an affinity with the enzyme–substrate-compounds and enzymes.

Regarding ZnO-NPs, an increased APX activity was caused by an increased H_2_O_2_, suggesting that pomegranate callus might adapt to NPs through an effective defense system. Moreover, this increased activity could be maintained through a high AsA concentration, as was observed in the previous study in a ZnO-NPs-treated pomegranate callus^4^. Likewise, García-López et al.^56^ found that ZnO-NPs caused a concentration-dependent elevation in the APX activity of the *Capsicum chinense* plants. In contrast, APX inhibition was noticed in the ZnO-BPs-treated callus. This shows that this enzyme may not be responsible for scavenging H_2_O_2_ in ZnO-BPs-treated calli or the H_2_O_2_ content associated with excess BPs was much less than that in the case of ZnONPs of callus cells. Accordingly, Wang et al.^57^ found that enhanced APX activity was noticed at low levels of ZnO (0.5–30 µg mL^−1^); however, it was significantly reduced at higher levels. Furthermore, in ZnO-NPs treatments, strong positive and negative relations between APX activity, Mn, and Fe, respectively, confirm that maintaining high levels of Mn may be required for AsA regeneration, and high levels of AsA may indicate an increased ROS production in iron-deprived callus tissues.

Moreover, it must be emphasized that at the low concentration of ZnO-NPs, an insignificant alter in K_m_ and the decrease in V_max_ of APX could result in the ZnO-NPs binding to the site other than the substrate-binding site. Conversely, the increase in K_cat_ and APX activity might indicate that the effect of ZnO-NPs at low concentrations on the enzyme was less than the formation of products. For the higher concentration of ZnO-NPs, the increase in K_m_ and the decrease in V_max_ of APX could result from the binding of ZnO-NPs to both the enzyme and the enzyme–substrate complex.

Similar to the low level of ZnO-NPs, the increase in K_cat_ can be suggested that the formation of the products was faster than the binding of ZnO-NPs with the enzyme and the enzyme–substrate complex. In the case of 10 μg mL^−1^ ZnO-BPs, the increase in the K_m_ value and an insignificant change in V_max_ may reveal that ZnO-BPs have an affinity for the active site of APX enzyme. The increase in the K_cat_ of APX enzyme under 10 μg mL^−1^ ZnO-BPs despite decreased activity indicates reduced product dissociation from the enzyme–substrate complex^54^. The increase of K_m_, V_max_, and K_cat_ of APX under 150 μg mL^−1^ ZnO-BPs indicate that the enzyme did not obey the Michaelis–Menten equation. These results indicate that ZnO-BPs at the high-level bind to the regulatory site causing the inactive form and reducing APX enzyme activity.

In this experiment, ZnO-NPs or ZnO-BPs treatments stimulated the PAL activity in pomegranate callus tissues. This result indicated that the PAL enzyme participates in the synthesis of secondary metabolites by stimulating the phenylpropanoid pathway in pomegranate calli, supporting antioxidant systems in relation to increased ROS, and resistance of calli against Zn stress. Under ZnO-NPs or BPs treatments, the increase in the total phenolics content resulting from PAL activity can act as an electron transmitter that catalyzes the transfer of electrons to ROS in the antioxidant system^4^. In line with these results, stimulation of the effect of PAL was observed in two wheat cultivars (cultivar Kıraç-66 and T. durum Desf. Cv. Kızıltan-91) under zinc stress^58^. The strong positive correlation between the PAL activity and the Mn concentration and the negative relationship between PAL and Fe confirms previous reports of Engelsma^59^ and Dixon and Paiva^60^ who concluded that PAL activity was stimulated by the presence of Mn and Fe deficiency.

The K_m_ and V_max_ of PAL were reduced by treating the pomegranate callus with ZnO-NPs, but K_cat_ increased, indicating an uncompetitive type inhibition with faster dissociation of products than the binding of ZnO-NPs to the enzyme–substrate complex. With regard to ZnO-BPs, the K_m_ decreased with increasing concentrations of ZnO-BPs, but V_max_ increased. The Michaelis–Menten equation was not realized to describe the relation between substrates concentration and the initial velocity of the reaction in the existence of ZnO-BPs^21^. An increase in K_cat_ indicates that a higher concentration of the substrate may overcome enzyme inhibition.

The result showed that the high level of ZnO-NPs significantly stimulated the PPO activity, while ZnO-BPs did not increase the activity significantly. This stimulation in PPO activity indicates that ZnO-NPs shift the callus metabolism toward the increased synthesis of phenolics, that was evident in our previous work^4^, and the activation of PPO; thus providing more antioxidants and defense against ZnO-NPs. Nonetheless, ZnO-BPs had no impact on the PPO activity and it reveals that the ROS content associated with ZnO-BPs was not high enough to stimulate all defense agents. Moreover, the stimulation of PPO activity, despite the decrease in copper reveals that the copper content did not decrease to the level affecting PPO synthesis. Likewise, the induction of PPO activity was observed in *Jatropha curcas* treated with 300 μmol L^−1^ Zn^61^.

A new observation was that under ZnO-NPs or BPs treatments, the reduction in K_m_, V_max_, and K_cat_ of the PPO enzyme may result from uncompetitive inhibition of ZnO. This finding indicates that ZnO-NPs or BPs may have a significant affinity towards the enzyme–substrate complex. However, under ZnO-NPs treatments, the increase in the PPO activity indicates that the effects of ZnO-NPs on product formation were greater than the interaction with the enzymatic substrate complex.

The entry of nanoparticles into plants is related to the zeta potential, and size^62^ that is able to enter from the apoplast and move to other parts via plasmodesmata^63^. ZnO-NPs or BPs treatments caused higher Zn concentrations in the pomegranate callus tissues, compared to controls, which mainly accumulated at 150 μg mL^−1^ of NPs approximately 1.1-fold higher than ZnO-BPs and confirmed that their uptake was obviously related to size and levels. These findings were also noted in previous investigations^64^, which indicated that small NPs produce new root openings due to their high surface interaction, causing an increased mineral flux.

Excess Zn may interfere with the absorption of other elements within pomegranate callus. ZnO-NPs or BPs treatments reduced the Cu, Fe, and the Mg content in callus tissues; whereas, it did not affect the K and P concentration. However, for Mn, an opposite position was observed, as it was increased with increasing concentrations of ZnO-NPs or BPs. These results indicate that zinc is transported along the symplastic-pathway and competes with copper, iron, and magnesium for similar transporters. In contrast, K, P, and Mn were absorbed in other ways that did not directly interfere with zinc. This suggestion was confirmed by the insignificant correlation between the K and Zn concentration in ZnO-NPs or BPs-treated calli. Thus, the present result is consistent with the previous results, which concluded that excess zinc had a more severe effect on micro-elements than on macro-nutrients in Arabidopsis^65^. Although the balance between zinc and phosphorus is related, and a decrease or increase of one element affects the other; however, with low zinc, there was an insignificant difference in the accumulation of phosphate transporter 1 transcripts of highly affinity phosphorus transporters within Arabidopsis roots^66^. The increase in manganese content was in line with earlier findings^67^. According to the negative strong correlations between the Mn and Fe contents under ZnO-NPs or BPs treatments, we speculated that the increase in Mn due to a decrease in Fe and their competition for the iron-had regulated transporter 1^68^.

Our results revealed that the activities of CAT, SOD, POD, APX, and PPO were associated with levels of ZnO-NPs and the high level of ZnO-BPs. These results indicate that the low level of ZnO-BPs did not induce a high production of ROS to bind to these enzymes, but that the high level of BPs and levels of NPs stimulated these enzymes to withstand ZnO stress. The macronutrients K, Mg, and the micronutrients Cu, Mn were also linked with ZnO-NPs and BPs. Moreover, the relationships between these nutrients indicate that zinc interferes with the uptake of these nutrients.

## Conclusion

Our results revealed that high doses of ZnO-NPs likely had the potential to enhance LOX, SOD, CAT, APX, PAL, and the PPO activity, but they reduced the POD activity. On the other hand, ZnO-BPs had no effect on LOX and PPO activity; whereas, they stimulated SOD, CAT, and PAL activity. When calli were exposed to ZnO-BPs, APX and POD activities decreased. Moreover, NPs resulted in mixed inhibition of both CAT and APX enzymes. Regarding BPs, they showed competitive inhibition of CAT, whereas APX and PAL enzymes did not undergo the Michael Menten equation. ZnO-NPs/BPs treatments may result from uncompetitive inhibition of POD, as well as product interaction with ZnO. Additionally, the results appeared that NPs or BPs may have a greater affinity for the PPO enzyme–substrate complexes and that the effects of NPs on product formation were greater than the interaction with the substrate–enzyme complexes. The accumulation of Zn in calli was proportional to the concentration of Zn in the medium, and this accumulation was higher in calli treated with ZnO-NPs than that treated with ZnO-BPs, which confirms the higher toxicity of NPs compared to BPs. Applications of NPs or BPs reduced the Cu, Fe, and the Mg content in callus tissues; whereas, they did not affect the K and P concentration. These results indicate that zinc is transported by the symplastic-pathway and competes with copper, iron, and magnesium for similar carriers. Our data unequivocally display that a low level of ZnO-BPs did not lead to a higher ROS production, whereas a higher level of BPs and levels of NPs boosted ROS production and interfered with the uptake of important nutrients, leading to an increase in some redox-enzymes to withstand ZnO stress. Our work contributes to the understanding of the effects of ZnO-NPs on the activities, the kinetics of redox-enzymes, and the interaction with nutrients, and additional testing is required.

## Supplementary information

Supplementary Information 1.
