# Supplementary material for: Impacts of zinc oxide nano and bulk particles on redox-enzymes of the Punica granatum callus
Source: Sci Rep. 2020 Nov 12;10:19722. doi: 10.1038/s41598-020-76664-4 (PMC7665012; doi:10.1038/s41598-020-76664-4)
Supplement: Supplementary file 1 — Supplementary Information 1. [file 41598_2020_76664_MOESM1_ESM.docx]

**Electric supplementary materials**

**Title: Impacts of zinc oxide nano and bulk particles on redox-enzymes of the *Punica granatum* callus**

Fatma A. Farghaly^1^, Abeer A. Radi^1^, Fatma A. Al-Kahtany^2^, and Afaf M. Hamada^1^

^1^Botany and Microbiology Department, Faculty of Science, Assiut University, Assiut 71516, Egypt

^2^Biology Department, Faculty of Science, Ibb University, Ibb, Yemen

**Journal:**

Scientific Reports

**Corresponding author:**

A.M. Hamada

Botany and Microbiology Department, Faculty of Science, Assiut University, Assiut, 71516, Egypt

Tel: +2-088-2412266

Fax: +2-088-2080209

E-mail: [afafhamada@yahoo.com](mailto:afafhamada@yahoo.com); [hamada@aun.edu.eg](mailto:hamada@aun.edu.eg)

**Content:**

**Fig. S1**

**Table S1–S12**

| **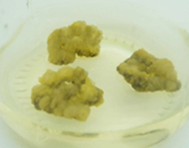** | **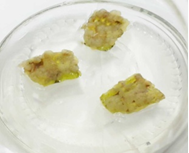** | **~~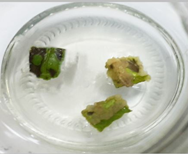~~** |
| --- | --- | --- |
| **0 µg mL^-1^ ZnO-NPs** | **10 µg mL^-1^ ZnO-NPs** | **150 µg mL^-1^ ZnO-NPs** |
| **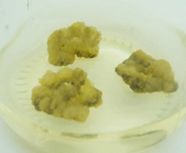** | **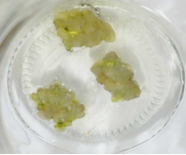** | **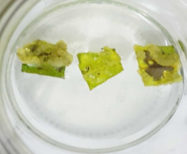** |
| **0 µg mL^-1^ ZnO-BPs** | **10 µg mL^-1^ ZnO-BPs** | **150 µg mL^-1^ ZnO-BPs** |
| **Fig. S1:** *Punica granatum* callus growth as influenced by different concentrations of ZnO-NPs and BPs (0, 10, and 150 μg mL^-1^) for 28 days. | | |

**Table S1:** Pearson correlation coefficients in ZnO-NPs treatments (the activity of antioxidant enzymes and minerals). Bivariate correlation analysis (n = 4). ** Correlation is significant at 0.01 level (two-tailed). * Correlation is significant at 0.05 (two-tailed) level.

|  | **LOX** | **SOD** | **CAT** | **POD** | **APX** | **PAL** | **PPO** | **Zn** | **K** | **Mg** | **P** | **Mn** | **Fe** | **Cu** |
| --- | --- | --- | --- | --- | --- | --- | --- | --- | --- | --- | --- | --- | --- | --- |
| **LOX** | 1 |  |  |  |  |  |  |  |  |  |  |  |  |  |
| **SOD** | 0.233 | 1 |  |  |  |  |  |  |  |  |  |  |  |  |
| **CAT** | 0.498 | 0.838** | 1 |  |  |  |  |  |  |  |  |  |  |  |
| **POD** | -0.323 | -0.844** | -0.931** | 1 |  |  |  |  |  |  |  |  |  |  |
| **APX** | 0.771** | 0.416 | 0.726** | -0.698** | 1 |  |  |  |  |  |  |  |  |  |
| **PAL** | 0.314 | 0.827** | 0.896** | -0.936** | 0.684** | 1 |  |  |  |  |  |  |  |  |
| **PPO** | 0.883** | 0.621* | 0.809** | -0.672* | 0.871** | 0.666* | 1 |  |  |  |  |  |  |  |
| **Zn** | 0.857** | 0.710* | 0.835** | -0.662 | 0.868** | 0.631 | 0.979** | 1 |  |  |  |  |  |  |
| **K** | -0.229 | -0.508 | -0.590 | 0.697* | -0.693* | -0.784* | -0.475 | -0.471 | 1 |  |  |  |  |  |
| **Mg** | -0.434 | -0.889** | -0.956** | 0.987** | -0.742* | -0.953** | -0.750* | -0.737* | 0.698* | 1 |  |  |  |  |
| **P** | 0.119 | 0.669* | 0.652 | -0.680* | 0.568 | 0.736* | 0.421 | 0.514 | -0.476 | -0.721* | 1 |  |  |  |
| **Mn** | 0.717* | 0.849** | 0.947** | -0.868** | 0.874** | 0.833** | 0.932** | 0.945** | -0.614 | -0.913** | 0.649 | 1 |  |  |
| **Fe** | -0.619 | -0.868** | -0.970** | 0.931** | -0.844** | -0.894** | -0.877** | -0.883** | 0.657 | 0.967** | -0.707* | -0.985** | 1 |  |
| **Cu** | -0.482 | -0.867** | -0.935** | 0.986** | -0.789* | -0.941** | -0.764* | -0.754* | 0.734* | 0.983** | -0.663 | -0.924** | 0.962** | 1 |

**LOX:** Lipoxygenase**; SOD:** Superoxide dismutase**; CAT:** Catalase**; POD:** Peroxidase**; APX:** Ascorbate peroxidase**, PAL:** Phenylalanine ammonia-lyase**; PPO:** Polyphenol oxidase

**Zn:** Zinc; **K:** Potassium; **Mg:** Magnesium; **P:** Phosphorus; **Mn:** Manganese; **Fe:** Iron; **Cu:** Copper

**Table S2:** Pearson correlation coefficients in ZnO-BPs treatments (the activity of antioxidant enzymes and minerals). Bivariate correlation analysis (n = 4). ** Correlation is significant at 0.01 level (two-tailed). * Correlation is significant at 0.05 (two-tailed) level.

|  | **LOX** | **SOD** | **CAT** | **POD** | **APX** | **PAL** | **PPO** | **Zn** | **K** | **Mg** | **P** | **Mn** | **Fe** | **Cu** |
| --- | --- | --- | --- | --- | --- | --- | --- | --- | --- | --- | --- | --- | --- | --- |
| **LOX** | 1 |  |  |  |  |  |  |  |  |  |  |  |  |  |
| **SOD** | -0.227 | 1 |  |  |  |  |  |  |  |  |  |  |  |  |
| **CAT** | -0.606* | 0.456 | 1 |  |  |  |  |  |  |  |  |  |  |  |
| **POD** | 0.276 | -0.809** | -0.794** | 1 |  |  |  |  |  |  |  |  |  |  |
| **APX** | 0.414 | -0.826** | -0.806** | 0.908** | 1 |  |  |  |  |  |  |  |  |  |
| **PAL** | -0.081 | 0.895** | 0.565* | -0.927** | -0.852** | 1 |  |  |  |  |  |  |  |  |
| **PPO** | -0.451 | 0.396 | 0.727** | -0.622* | -0.739** | 0.394 | 1 |  |  |  |  |  |  |  |
| **Zn** | -0.552 | 0.448 | 0.989** | -0.794* | -0.826** | 0.573 | 0.949** | 1 |  |  |  |  |  |  |
| **K** | 0.071 | -0.774* | -0.560 | 0.798** | 0.759* | -0.812** | -0.720* | -0.588 | 1 |  |  |  |  |  |
| **Mg** | 0.312 | -0.743* | -0.857** | 0.972** | 0.975** | -0.876** | -0.934** | -0.890** | 0.761* | 1 |  |  |  |  |
| **P** | -0.403 | 0.324 | 0.726* | -0.534 | -0.664 | 0.419 | 0.601 | 0.766* | -0.290 | -0.680* | 1 |  |  |  |
| **Mn** | -0.456 | 0.678* | 0.921** | -0.913** | -0.937** | 0.763* | 0.982** | 0.951** | -0.689* | -0.972** | 0.695* | 1 |  |  |
| **Fe** | 0.621 | -0.407 | -0.991** | 0.749* | 0.784* | -0.505 | -0.928** | -0.992** | 0.553 | 0.847** | -0.758* | -0.925** | 1 |  |
| **Cu** | 0.1642 | -0.797* | -0.740* | 0.993** | 0.948** | -0.930** | -0.884** | -0.772* | 0.823** | 0.960** | -0.482 | -0.904** | 0.724* | 1 |

**LOX:** Lipoxygenase**; SOD:** Superoxide dismutase**; CAT:** Catalase**; POD:** Peroxidase**; APX:** Ascorbate peroxidase**, PAL:** Phenylalanine ammonia-lyase**; PPO:** Polyphenol oxidase

**Zn:** Zinc; **K:** Potassium; **Mg:** Magnesium; **P:** Phosphorus; **Mn:** Manganese; **Fe:** Iron; **Cu:** Copper

**Table S3:** Pearson correlation coefficients in ZnO-NPs treatments [the kinetics activity of catalase (CAT) and minerals]. Bivariate correlations analysis (n = 4). ** Correlation is significant at 0.01 level (two-tailed). * Correlation is significant at 0.05 (two-tailed) level

|  | **CAT K_m_** | **CAT V_m_** | **CAT K_cat_** | **Zn** | **K** | **Mg** | **P** | **Mn** | **Fe** | **Cu** |
| --- | --- | --- | --- | --- | --- | --- | --- | --- | --- | --- |
| **CAT K_m_** | 1 |  |  |  |  |  |  |  |  |  |
| **CAT V_m_** | -0.253 | 1 |  |  |  |  |  |  |  |  |
| **CAT K_cat_** | 0.829** | 0.323 | 1 |  |  |  |  |  |  |  |
| **Zn** | 0.963** | -0.017 | 0.948** | 1 |  |  |  |  |  |  |
| **K** | -0.591 | 0.542 | -0.296 | -0.471 | 1 |  |  |  |  |  |
| **Mg** | -0.892** | 0.684* | -0.497 | -0.737* | 0.698* | 1 |  |  |  |  |
| **P** | 0.639 | -0.542 | 0.334 | 0.514 | -0.476 | -0.721* | 1 |  |  |  |
| **Mn** | 0.996** | -0.336 | 0.799** | 0.945** | -0.614 | -0.913** | 0.649 | 1 |  |  |
| **Fe** | -0.975** | 0.482 | -0.696* | -0.883** | 0.658 | 0.967** | -0.707* | -0.985** | 1 |  |
| **Cu** | -0.896** | 0.642 | -0.527 | -0.754* | 0.734* | 0.983** | -0.663 | -0.924** | 0.962** | 1 |

**K_m_:** Michaelis constant; **V_m_:** Maximum velocity; **K_cat_:** Catalytic rate constant

**Zn:** Zinc; **K:** Potassium; **Mg:** Magnesium; **P:** Phosphorus; **Mn:** Manganese; **Fe:** Iron; **Cu:** Copper

**Table S4:** Pearson correlation coefficients in ZnO-BPs treatments [the kinetics activity of catalase (CAT) and minerals]. Bivariate correlations analysis (n = 4). ** Correlation is significant at 0.01 level (two-tailed). * Correlation is significant at 0.05 (two-tailed) level.

|  | **CAT K_m_** | **CAT V_m_** | **CAT K_cat_** | **Zn** | **K** | **Mg** | **P** | **Mn** | **Fe** | **Cu** |
| --- | --- | --- | --- | --- | --- | --- | --- | --- | --- | --- |
| **CAT K_m_** | 1 |  |  |  |  |  |  |  |  |  |
| **CAT V_m_** | -0.841** | 1 |  |  |  |  |  |  |  |  |
| **CAT K_cat_** | 0.866** | -0.501 | 1 |  |  |  |  |  |  |  |
| **Zn** | 0.245 | 0.165 | 0.711* | 1 |  |  |  |  |  |  |
| **K** | -0.699* | 0.419 | -0.820** | -0.588 | 1 |  |  |  |  |  |
| **Mg** | -0.651 | 0.283 | -0.941** | -0.890** | 0.761* | 1 |  |  |  |  |
| **P** | 0.142 | 0.118 | 0.510 | 0.766* | -0.290 | -0.680* | 1 |  |  |  |
| **Mn** | 0.491 | -0.107 | 0.855** | 0.951** | -0.689* | -0.972** | 0.695* | 1 |  |  |
| **Fe** | -0.174 | -0.239 | -0.662 | -0.992** | 0.553 | 0.847** | -0.758* | -0.925** | 1 |  |
| **Cu** | -0.766* | 0.448 | -0.963** | -0.772* | 0.823** | 0.960** | -0.482 | -0.904** | 0.724* | 1 |

**K_m_:** Michaelis constant; **V_m_:** Maximum velocity; **K_cat_:** Catalytic rate constant

**Zn:** Zinc; **K:** Potassium; **Mg:** Magnesium; **P:** Phosphorus; **Mn:** Manganese; **Fe:** Iron; **Cu:** Copper

**Table S5:** Pearson correlation coefficients in ZnO-NPs treatments [the kinetics activity of peroxidase (POD) and minerals]. Bivariate correlations analysis (n = 4). ** Correlation is significant at 0.01 level (two-tailed). * Correlation is significant at 0.05 (two-tailed) level.

|  | **POD K_m_** | **POD V_m_** | **POD K_cat_** | **Zn** | **K** | **Mg** | **P** | **Mn** | **Fe** | **Cu** |
| --- | --- | --- | --- | --- | --- | --- | --- | --- | --- | --- |
| **POD K_m_** | 1 |  |  |  |  |  |  |  |  |  |
| **POD V_m_** | 0.986** | 1 |  |  |  |  |  |  |  |  |
| **POD K_cat_** | -0.587* | -0.441 | 1 |  |  |  |  |  |  |  |
| **Zn** | -0.317 | -0.156 | 0.942** | 1 |  |  |  |  |  |  |
| **K** | 0.659 | 0.610 | -0.611 | -0.471 | 1 |  |  |  |  |  |
| **Mg** | 0.871** | 0.779* | -0.918** | -0.737* | 0.698* | 1 |  |  |  |  |
| **P** | -0.686* | -0.625 | 0.648 | 0.514 | -0.476 | -0.721* | 1 |  |  |  |
| **Mn** | -0.604 | -0.464 | 0.997** | 0.945** | -0.614 | -0.913** | 0.649 | 1 |  |  |
| **Fe** | 0.725* | 0.601 | -0.988** | -0.883** | 0.657 | 0.967** | -0.707* | -0.985** | 1 |  |
| **Cu** | 0.838** | 0.741* | -0.920** | -0.754* | 0.734* | 0.983** | -0.663 | -0.924** | 0.962** | 1 |

**K_m_:** Michaelis constant; **V_m_:** Maximum velocity; **K_cat_:** Catalytic rate constant

**Zn:** Zinc; **K:** Potassium; **Mg:** Magnesium; **P:** Phosphorus; **Mn:** Manganese; **Fe:** Iron; **Cu:** Copper

**Table S6:** Pearson correlation coefficients in ZnO-BPs treatments [the kinetics activity of peroxidase (POD) and minerals]. Bivariate correlations analysis (n = 4). ** Correlation is significant at 0.01 level (two-tailed). * Correlation is significant at 0.05 (two-tailed) level.

|  | **POD K_m_** | **POD V_m_** | **POD K_cat_** | **Zn** | **K** | **Mg** | **P** | **Mn** | **Fe** | **Cu** |
| --- | --- | --- | --- | --- | --- | --- | --- | --- | --- | --- |
| **POD K_m_** | 1 |  |  |  |  |  |  |  |  |  |
| **POD V_m_** | -0.370 | 1 |  |  |  |  |  |  |  |  |
| **POD K_cat_** | -0.949** | 0.509 | 1 |  |  |  |  |  |  |  |
| **Zn** | -0.959** | 0.079 | 0.923** | 1 |  |  |  |  |  |  |
| **K** | 0.409 | 0.516 | -0.309 | -0.588 | 1 |  |  |  |  |  |
| **Mg** | 0.730* | 0.372 | -0.657 | -0.890** | 0.761* | 1 |  |  |  |  |
| **P** | -0.737* | 0.084 | 0.728* | 0.766* | -0.290 | -0.680* | 1 |  |  |  |
| **Mn** | -0.834** | -0.186 | 0.784* | 0.951** | -0.689* | -0.972** | 0.695* | 1 |  |  |
| **Fe** | 0.969** | -0.147 | -0.939** | -0.992** | 0.553 | 0.847** | -0.758* | -0.925** | 1 |  |
| **Cu** | 0.573 | 0.532 | -0.492 | -0.772* | 0.823** | 0.960** | -0.482 | -0.904** | 0.724* | 1 |

**K_m_:** Michaelis constant; **V_m_:** Maximum velocity; **K_cat_:** Catalytic rate constant

**Zn:** Zinc; **K:** Potassium; **Mg:** Magnesium; **P:** Phosphorus; **Mn:** Manganese; **Fe:** Iron; **Cu:** Copper

**Table S7:** Pearson correlation coefficients in ZnO-NPs treatments [the kinetics activity of ascorbate peroxidase (APX) and minerals]. Bivariate correlations analysis (n = 4). ** Correlation is significant at 0.01 level (two-tailed). * Correlation is significant at 0.05 (two-tailed) level.

|  | **APX K_m_** | **APX V_m_** | **APX K_cat_** | **Zn** | **K** | **Mg** | **P** | **Mn** | **Fe** | **Cu** |
| --- | --- | --- | --- | --- | --- | --- | --- | --- | --- | --- |
| **APX K_m_** | 1 |  |  |  |  |  |  |  |  |  |
| **APX V_m_** | -0.022 | 1 |  |  |  |  |  |  |  |  |
| **APX K_cat_** | 0.224 | -0.875** | 1 |  |  |  |  |  |  |  |
| **Zn** | 0.664 | -0.486 | 0.620 | 1 |  |  |  |  |  |  |
| **K** | 0.148 | 0.636 | -0.754* | -0.471 | 1 |  |  |  |  |  |
| **Mg** | -0.055 | 0.932** | -0.935** | -0.737* | 0.698* | 1 |  |  |  |  |
| **P** | 0.017 | -0.732* | 0.668* | 0.514 | -0.476 | -0.721* | 1 |  |  |  |
| **Mn** | 0.417 | -0.736* | 0.811** | 0.945** | -0.614 | -0.913** | 0.649 | 1 |  |  |
| **Fe** | -0.275 | 0.835** | -0.872** | -0.883** | 0.657 | 0.967** | -0.707* | -0.985** | 1 |  |
| **Cu** | -0.071 | 0.910** | -0.910** | -0.754* | 0.734* | 0.983** | -0.663 | -0.924** | 0.962** | 1 |

**K_m_:** Michaelis constant; **V_m_:** Maximum velocity; **K_cat_:** Catalytic rate constant

**Zn:** Zinc; **K:** Potassium; **Mg:** Magnesium; **P:** Phosphorus; **Mn:** Manganese; **Fe:** Iron; **Cu:** Copper

**Table S8:** Pearson correlation coefficients in ZnO-BPs treatments [the kinetics activity of ascorbate peroxidase (APX) and minerals]. Bivariate correlations analysis (n = 4). ** Correlation is significant at 0.01 level (two-tailed). * Correlation is significant at 0.05 (two-tailed) level.

|  | **APX K_m_** | **APX V_m_** | **APX K_cat_** | **Zn** | **K** | **Mg** | **P** | **Mn** | **Fe** | **Cu** |
| --- | --- | --- | --- | --- | --- | --- | --- | --- | --- | --- |
| **APX K_m_** | 1 |  |  |  |  |  |  |  |  |  |
| **APX V_m_** | 0.867** | 1 |  |  |  |  |  |  |  |  |
| **APX K_cat_** | 0.996** | 0.836** | 1 |  |  |  |  |  |  |  |
| **Zn** | 0.974** | 0.957** | 0.953** | 1 |  |  |  |  |  |  |
| **K** | -0.688* | -0.433 | -0.736* | -0.588 | 1 |  |  |  |  |  |
| **Mg** | -0.969** | -0.728* | -0.981** | -0.890** | 0.761* | 1 |  |  |  |  |
| **P** | 0.739* | 0.722* | 0.715* | 0.766* | -0.290 | -0.680* | 1 |  |  |  |
| **Mn** | 0.987** | 0.836** | 0.986** | 0.951** | -0.689* | -0.972** | 0.695* | 1 |  |  |
| **Fe** | -0.950** | -0.961** | -0.920** | -0.992** | 0.553 | 0.847** | -0.758* | -0.925** | 1 |  |
| **Cu** | -0.889** | -0.573 | -0.914** | -0.772* | 0.823** | 0.960** | -0.482 | -0.904** | 0.724* | 1 |

**K_m_:** Michaelis constant; **V_m_:** Maximum velocity; **K_cat_:** Catalytic rate constant

**Zn:** Zinc; **K:** Potassium; **Mg:** Magnesium; **P:** Phosphorus; **Mn:** Manganese; **Fe:** Iron; **Cu:** Copper

**Table S9:** Pearson correlation coefficients in ZnO-NPs treatments [the kinetics activity of phenylalanine ammonia-lyase (PAL) and minerals]. Bivariate correlations analysis (n = 4). ** Correlation is significant at 0.01 level (two-tailed). * Correlation is significant at 0.05 (two-tailed) level.

|  | **PAL K_m_** | **PAL V_m_** | **PAL K_cat_** | **Zn** | **K** | **Mg** | **P** | **Mn** | **Fe** | **Cu** |
| --- | --- | --- | --- | --- | --- | --- | --- | --- | --- | --- |
| **PAL K_m_** | 1 |  |  |  |  |  |  |  |  |  |
| **PAL V_m_** | 0.904** | 1 |  |  |  |  |  |  |  |  |
| **PAL K_cat_** | -0.982** | -0.807** | 1 |  |  |  |  |  |  |  |
| **Zn** | -0.529 | -0.824** | 0.376 | 1 |  |  |  |  |  |  |
| **K** | 0.703* | 0.736* | -0.656 | -0.471 | 1 |  |  |  |  |  |
| **Mg** | 0.960** | 0.968** | -0.901** | -0.737* | 0.698* | 1 |  |  |  |  |
| **P** | -0.739* | -0.736* | 0.691* | 0.514 | -0.476 | -0.721* | 1 |  |  |  |
| **Mn** | -0.771* | -0.945** | 0.654 | 0.945** | -0.614 | -0.913** | 0.649 | 1 |  |  |
| **Fe** | 0.864** | 0.978** | -0.767* | -0.883** | 0.657 | 0.967** | -0.707* | -0.985** | 1 |  |
| **Cu** | 0.930** | 0.945** | -0.872** | -0.754* | 0.734* | 0.983** | -0.663 | -0.924** | 0.962** | 1 |

**K_m_:** Michaelis constant; **V_m_:** Maximum velocity; **K_cat_:** Catalytic rate constant

**Zn:** Zinc; **K:** Potassium; **Mg:** Magnesium; **P:** Phosphorus; **Mn:** Manganese; **Fe:** Iron; **Cu:** Copper

**Table S10:** Pearson correlation coefficients in ZnO-BPs treatments [the kinetics activity of phenylalanine ammonia-lyase (PAL) and minerals]. Bivariate correlations analysis (n = 4). ** Correlation is significant at 0.01 level (two-tailed). * Correlation is significant at 0.05 (two-tailed) level.

|  | **PAL K_m_** | **PAL V_m_** | **PAL K_cat_** | **Zn** | **K** | **Mg** | **P** | **Mn** | **Fe** | **Cu** |
| --- | --- | --- | --- | --- | --- | --- | --- | --- | --- | --- |
| **PAL K_m_** | 1 |  |  |  |  |  |  |  |  |  |
| **PAL V_m_** | -0.917** | 1 |  |  |  |  |  |  |  |  |
| **PAL K_cat_** | -0.993** | 0.967** | 1 |  |  |  |  |  |  |  |
| **Zn** | -0.790* | 0.967** | 0.723* | 1 |  |  |  |  |  |  |
| **K** | 0.798** | -0.696* | -0.802** | -0.588 | 1 |  |  |  |  |  |
| **Mg** | 0.977** | -0.974** | -0.952** | -0.890** | 0.761* | 1 |  |  |  |  |
| **P** | -0.601 | 0.729* | 0.538 | 0.766* | -0.290 | -0.680* | 1 |  |  |  |
| **Mn** | -0.913** | 0.989** | 0.873** | 0.951** | -0.689* | -0.972** | 0.695* | 1 |  |  |
| **Fe** | 0.739* | -0.943** | -0.669* | -0.992** | 0.553 | 0.847** | -0.758* | -0.925** | 1 |  |
| **Cu** | 0.973** | -0.904** | -0.975** | -0.772* | 0.823** | 0.960** | -0.482 | -0.904** | 0.724* | 1 |

**K_m_:** Michaelis constant; **V_m_:** Maximum velocity; **K_cat_:** Catalytic rate constant

**Zn:** Zinc; **K:** Potassium; **Mg:** Magnesium; **P:** Phosphorus; **Mn:** Manganese; **Fe:** Iron; **Cu:** Copper

**Table S11:** Pearson correlation coefficients in ZnO-NPs treatments [the kinetics activity of polyphenol oxidase (PPO) and minerals]. Bivariate correlations analysis (n = 4). ** Correlation is significant at 0.01 level (two-tailed). * Correlation is significant at 0.05 (two-tailed) level.

|  | **PPO K_m_** | **PPO V_m_** | **PPO K_cat_** | **Zn** | **K** | **Mg** | **P** | **Mn** | **Fe** | **Cu** |
| --- | --- | --- | --- | --- | --- | --- | --- | --- | --- | --- |
| **PPO K_m_** | 1 |  |  |  |  |  |  |  |  |  |
| **PPO V_m_** | 0.999** | 1 |  |  |  |  |  |  |  |  |
| **PPO K_cat_** | 0.967** | 0.979** | 1 |  |  |  |  |  |  |  |
| **Zn** | -0.501 | -0.453 | -0.262 | 1 |  |  |  |  |  |  |
| **K** | 0.707* | 0.699* | 0.651 | -0.471 | 1 |  |  |  |  |  |
| **Mg** | 0.952** | 0.934** | 0.843** | -0.737* | 0.698* | 1 |  |  |  |  |
| **P** | -0.715* | -0.706* | -0.645 | 0.514 | -0.476 | -0.721* | 1 |  |  |  |
| **Mn** | -0.752* | -0.715* | -0.558 | 0.945** | -0.614 | -0.913** | 0.649 | 1 |  |  |
| **Fe** | 0.848** | 0.818** | 0.684* | -0.883** | 0.657 | 0.967** | -0.707* | -0.985** | 1 |  |
| **Cu** | 0.929** | 0.909** | 0.812** | -0.754* | 0.734* | 0.983** | -0.663 | -0.924** | 0.962** | 1 |

**K_m_:** Michaelis constant; **V_m_:** Maximum velocity; **K_cat_:** Catalytic rate constant

**Zn:** Zinc; **K:** Potassium; **Mg:** Magnesium; **P:** Phosphorus; **Mn:** Manganese; **Fe:** Iron; **Cu:** Copper

**Table S12:** Pearson correlation coefficients in ZnO-BPs treatments [the kinetics activity of polyphenol oxidase (PPO) and minerals]. Bivariate correlations analysis (n = 4). ** Correlation is significant at 0.01 level (two-tailed). * Correlation is significant at 0.05 (two-tailed) level.

|  | **PPO K_m_** | **PPO V_m_** | **PPO K_cat_** | **Zn** | **K** | **Mg** | **P** | **Mn** | **Fe** | **Cu** |
| --- | --- | --- | --- | --- | --- | --- | --- | --- | --- | --- |
| **PPO K_m_** | 1 |  |  |  |  |  |  |  |  |  |
| **PPO V_m_** | 0.949** | 1 |  |  |  |  |  |  |  |  |
| **PPO K_cat_** | 0.938** | 0.930** | 1 |  |  |  |  |  |  |  |
| **Zn** | -0.194 | -0.352 | 0.008 | 1 |  |  |  |  |  |  |
| **K** | 0.724* | 0.716* | 0.490 | -0.588 | 1 |  |  |  |  |  |
| **Mg** | 0.603 | 0.730* | 0.424 | -0.890** | 0.761* | 1 |  |  |  |  |
| **P** | -0.029 | -0.231 | 0.086 | 0.766* | -0.290 | -0.680* | 1 |  |  |  |
| **Mn** | -0.448 | -0.582 | -0.244 | 0.951** | -0.689* | -0.972** | 0.695* | 1 |  |  |
| **Fe** | 0.131 | 0.280 | -0.089 | -0.992** | 0.553 | 0.847** | -0.758* | -0.925** | 1 |  |
| **Cu** | 0.757* | 0.839** | 0.596 | -0.772* | 0.823** | 0.960** | -0.482 | -0.904** | 0.724* | 1 |

**K_m_:** Michaelis constant; **V_m_:** Maximum velocity; **K_cat_:** Catalytic rate constant

**Zn:** Zinc; **K:** Potassium; **Mg:** Magnesium; **P:** Phosphorus; **Mn:** Manganese; **Fe:** Iron; **Cu:** Copper
